# Retinal Degeneration Caused by Ago2 Disruption

**DOI:** 10.1167/iovs.62.12.14

**Published:** 2021-09-16

**Authors:** Xue-Jiao Chen, Chang-Jun Zhang, Ya-Han Wang, Zi-Bing Jin

**Affiliations:** 1 School of Ophthalmology & Optometry and Eye Hospital, Wenzhou Medical University, Wenzhou, China; 2 Beijing Institute of Ophthalmology, Beijing Tongren Eye Center, Beijing Tongren Hospital, Capital Medical University, Beijing Ophthalmology & Visual Science Key Laboratory, Beijing, China; 3 School of Basic Medical Sciences, Wenzhou Medical University, Wenzhou, China

**Keywords:** Ago2, retina degeneration, noncoding RNAs, regulation

## Abstract

**Purpose:**

Argonaute proteins are key players in small RNA-guided gene silencing processes. Ago2 is the member of the Argonaute subfamily with slicer endonuclease activity and is critical for microRNA homeostasis and indispensable for biological development. However, the impact of Ago2 dysregulation in the retina remains to be fully explored. In this study, we studied the role of Ago2 in mouse retina.

**Methods:**

We explored the function of Ago2 in the mouse retina through an adeno-associated virus-mediated *Ago2* disruption mouse model. An ERG was carried out to determine the retinal function. Spectral domain optical coherence tomography, fundus photographs, and immunostaining were performed to investigate the retinal structure. A quantitative RT-PCR assay was used to determine the expression of noncoding RNAs.

**Results:**

Both silencing and overexpression of Ago2 in mouse retina resulted in significant retinal morphological alterations and severe impairment of retinal function, mainly with a thinned outer nuclear layer, shortened inner segment/outer segment, and diminished ERG responses. Furthermore, Ago2 disruption resulted in alterations of noncoding RNAs in retina.

**Conclusions:**

Our finding demonstrated that Ago2 interruption led to severe retinal degeneration, suggested that Ago2 homeostasis contributed to retinal structural and functional maintenance.

Small RNA-guided gene silencing is an essential biological regulatory process in all cells, and the major proteins in this process are members of the Argonaute proteins. As types of small RNAs, microRNAs (miRNAs), short interfering RNAs (siRNA) and PIWI-interacting RNAs typically assemble with Argonaute proteins into the RNA-induced silencing complex (RISC).^1^^–^^3^ As the components of RISC, Argonaute proteins have unique biochemical and structured properties.^4^^,^^5^ The Argonaute family can be divided into Ago proteins and PIWI proteins. Ago proteins mainly interact with miRNAs or siRNAs and coordinate downstream gene-silencing events in cytoplasm. PIWI proteins mainly bind to PIWI-interacting RNAs and function in germline cells.^3^

It has been reported that the RISC assembly of miRNAs and siRNAs share a similar manner.^6^ miRNAs, whose length is approximately 22 nt, regulate a large variety of biological processes by a post-transcriptional gene silencing mechanism. DiGeorge syndrome critical region 8 (Dgcr8) and Drosha process primary miRNAs into short hairpins called precursor miRNAs (pre-miRNAs). The pre-miRNAs are further processed by Dicer into mature miRNAs.^7^ Ago proteins (Ago1–4) directly bind to mature miRNAs in RISC and carry out post-transcriptional gene silencing of protein-coding genes.^2^^,^^3^ The siRNAs, whose length is approximately 21 nt, are widely used as a useful tool to knock down genes of interest.^8^ siRNAs are processed from long double stranded RNA and assemble with Ago proteins into RISC to mediate gene silencing.^9^

Ago2 possess catalytic activity and contributes “slicer” activity to RISC.^10^ It is a key player in RISC and a master regulator of miRNA genesis as well as functionality, and provides the catalytic engine for RNA interference. It has been shown that only Ago2 is required for mouse development and disruption of Ago2 through a targeted insertional mutagenesis strategy produced an embryonic-lethal phenotype.^11^ In another study, Ago2-deficient embryos exhibited developmental arrest caused at the post-transcriptional level via the small RNA–protein complex.^12^ Ago2-deficient oocytes have severe defects in spindle formation and chromosome arrangement, similar to the abnormalities in Dicer-deficient oocytes. A comparison of gene expression profiles in Ago2 and Dicer knockout (KO) oocytes revealed that Ago2 and Dicer have distinct roles.^12^ In addition, another group showed that the slicer endonuclease activity of Ago2 is dispensable for hematopoiesis. They also confirmed that other Ago proteins seem dispensable for mammalian development and cannot compensate for the deletion of Ago2.^13^

The retina, a key part of the central nervous system, is critical in transducing light into electrophysiological signals that ultimately form vision. The miRNA-guided gene silencing is essential for retinal development, maintenance and homeostasis.^14^^,^^15^ Drosha/Dgcr8, Dicer, and Agos are central factors in miRNA biogenesis and function.^6^ And the essential roles of Drosha/Dgcr8 and Dicer in retina have been well-elaborated.^16^^–^^22^ However, the regulation of Agos in the retina remains unknown and needs to be explored.

As the widely studied subclass of noncoding RNAs, miRNAs has been well-characterized in retina, including *miR-183/96/182* (*miR-183C*),^23^^–^^26^ *miR-204*,^27^^,^^28^ *miR-124a*,^29^ *miR-9*, and *miR-23a*.^30^^,^^31^ However, only a handful of long noncodings RNAs (lncRNAs), whose length were more than 200 nucleotides, has been investigated in the retina. Taurine upregulated gene (*Tug1*) was the first reported lncRNA in retina,^32^ followed by retinal noncoding RNA 2 (*Rncr2*)^33^^,^^34^; *Six3os* and *Vax2os1* were also the lncRNAs that received the early attention.^35^ *Meg3*, once implicated in cancers, has been reported to be linked to the retina.^36^ Whether Ago2 interruption affects these noncoding RNAs in retina also need to explore.

Ago2 has been reported to be a candidate master regulator during miRNA biogenesis^37^ and miRNA-mediated repression of target genes.^1^^,^^2^ Previous studies have found that dysregulation of genes^16^^–^^22^ in miRNA biogenesis and miRNAs expressed in retina^23^^–^^31^ can be detrimental to retina. Meanwhile, the overexpression of Ago2 protein in cells could increase the abundance of mature miRNAs, more Ago2 could bind and stabilize mature miRNAs, and thereby enhance miRNAs abundance and guide more mRNAs silencing.^37^ We hypothesized that silencing or overexpression of Ago2 in retina would result in dysregulation of miRNAs and miRNA targets, and then lead to retinal degeneration.

Herein, to elucidate the regulatory function of Ago2 in the retina, we used adeno-associated virus (AAV)-mediated interference of Ago2 in vivo. We found that Ago2 disruption led to severe retinal degeneration, including shortened inner segment/outer segment (IS/OS), thinned outer nuclear layer (ONL), and impaired ERG response. Moreover, some noncoding RNAs expressed in retina were dysregulated. Our results indicate that Ago2 is indispensable for retinal structure and functional maintenance.

## Methods

### Animals and Ethics Statement

C57BL/6J mice were bred in the animal facility of Ophthalmology & Optometry in Wenzhou Medical University and were maintained under a 12-hour light–dark cycle and had free access to food and water. All experiments were carried out according to the ARVO Statement.

### AAV-Mediated *Ago2* Disruption

The silencing and overexpression of Ago2 in mouse retina has been achieved through subretinal injection of AAV-sh*Ago2*-EGFP and AAV-*Ago2*-3Flag vectors. To generate the AAV-sh*Ago2*-EGFP, the target sequence GCACACGCTCTGTGTCAAT was cloned into the GV478 vector containing pU6-MCS-CAG-EGFP. The control AAV-EGFP was the GV478 vector with nonsense sequence CGCTGAGTACTTCGAAATGTC. To generate AAV-*Ago2*-3Flag, the target *Ago2* sequence (2622 bp) was amplified by primer TACCGGACTCAGATCTCGAGATGTACTCGGGAGC-CGGCCCCGTTC and GATCCCGGGCCCGCGGTACCGTAGC-AAAGTACATGGTGCGCAGTGTG, then, the PCR product was cloned into the Xho I and Kpn I sites of a GV411 vector containing pCMV-betaGlobin-MCS-3Flag-SV40 PolyA. The GV411 empty vector with no target sequence was used as control AAV-3Flag. The titers of AAV-sh*Ago2*-EGFP and AAV-EGFP were 1.23 × 10^12^ and 1.30 × 10^12^ TU/mL, respectively. The titers of AAV-3Flag and AAV-*Ago2*-3Flag were 1.09 × 10^12^ and 1.08 × 10^12^ TU/mL, respectively.

### AAV Subretinal and Intravitreal Injections

The subretinal injection of AAV was performed as previously described.^23^^,^^38^ P30 C57BL/6J mice were used for AAV injection. First, 0.5% tropicamide was used to dilate the pupil of the mouse for 10 minutes. Second, mice were anesthetized with pentobarbital sodium. Third, both eyes of the mice were covered with ofloxacin eye cream to avoid infection and improve conjunction with the corneal electrode. Then, the surgery was performed under an operating microscope. A small incision was made in the cornea near the sclera with a sharp 30-G hypodermic needle. Then, 1 µL of AAV-sh*Ago2*-EGFP (1.23 × 10^13^ TU/mL)/AAV-*Ago2*-3Flag (1.08 × 10^13^ TU/mL) for the right eyes and an equal dose of the control vector AAV-EGFP/AAV-3Flag for the left eyes were injected slowly into the subretinal or intravitreal space using a blunt 5-µL Hamilton syringe held in a micromanipulator. All injections were performed bilaterally. For the subretinal injection, the presence of a retinal detachment indicated the successful injection.

### Fundus Photography and SD-OCT

AAV-sh*Ago2*-EGFP/AAV-EGFP and AAV-*Ago2*-3Flag/AAV-3Flag injected mice were dilated with 0.5% tropicamide and anesthetized using pentobarbital sodium. Fundus photography and SD-OCT were then performed. For SD-OCT measurements, images crossing through the optic nerve were obtained and collected for each eye. The thicknesses of the different retinal layers were measured using Insight software (Pleasanton, CA).

### Focal ERG

Scotopic and photopic responses in both eyes of the mouse were recorded with a Phoenix Micron IV as previously described. Briefly, mice were dark-adapted overnight and pupils were dilated using 0.5% tropicamide. Then, the mice were anesthetized intraperitoneally with pentobarbital sodium. The ground electrodes and referential needle were punctured into the tail and cheek, respectively. Scotopic ERG was recorded at −1.7 to 3.1 log cd-s/m^2^ stimulus intensity with different interstimulus intervals. Photopic ERG was measured at 0.7 to 3.3 log cd-s/m^2^ with different interstimulus intervals after 10 minutes of light adaptation with a background illumination of 5.0 log cd/m^2^.

### Retinal Immunostaining

Whole eyeballs of mice were isolated and fixed in 4% (wt/vol) paraformaldehyde in PBS for 20 minutes. Retinas were dissected and refixed with 4% (wt/vol) paraformaldehyde for an extra 15 minutes. Retinas were then dehydrated in 30% (wt/vol) sucrose and embedded in embedding medium (Neg-50, Thermo). 12-µm-thick cryosection slides were obtained and stored at −80 °C. For immunostaining, cryosection slides were placed at room temperature, washed with PBS, blocked in blocking buffer (4% BSA, 0.5% Triton X-100 in PBS) for 45 minutes, treated with primary antibody at 4 °C overnight, and then incubated with secondary antibody at room temperature for 1 hour. Then primary and secondary antibodies were used as follows: rabbit anti-Cone arrestin (1:50, Millipore, AB15282), mouse anti-Rhodopsin (1:500, Sigma, O4886), rabbit anti-Recoverin (1: 500, Millipore, AB5585), rabbit anti-Pkcα (1:100, Sigma, P4334), rabbit anti–glutamine synthetase (1:200, Abcam, ab73593), mouse anti–glial fibrillary acidic protein (1:200, Santacruz, sc-33673), mouse anti–cellular retinaldehyde binding protein (1:200, Abcam, ab15051), mouse anti-GFP (1:200, Invitrogen, 3E6), goat anti-Flag (1:200, Abcam, ab1275), and guinea pig anti-vGlut1 (1:100, Millipore, AB5905). Donkey anti-rabbit IgG conjugated to Alexa Fluor 488 (1:200, Life Technologies), donkey anti-mouse IgG conjugated to Alexa Fluor 594 (1:200, Li-cor Biosciences), Donkey anti-rabbit IgG conjugated to Alexa Fluor 680 (1:200, Life Technologies), and goat anti-guinea pig IgG conjugated to Alexa Fluor 568 (1:200, Abcam) were used as secondary antibodies. Samples were stained with 4,6-diamidino-2-phenylindole and visualized using laser-scanning confocal microscope (TCS SP8, Leica). Images were taken at 200 to 400 µm location in reference to the optic nerve.

### RNA Isolation and Quantitative RT-PCR (qRT-PCR)

To harvest retinal RNA, eyeballs were isolated from mice treated with AAV-sh*Ago2*-EGFP/AAV-EGFP and AAV-*Ago2*-3Flag/AAV-3Flag. Retinas were dissected and placed in TRIzol reagent. RNA was extracted using the RNeasy Mini Kit (Qiagen). For *miR-183C*, cDNA was synthesized using 1 µg of total RNA, TaqMan miRNA RT probe (The assay ID of *miR-183-5p*, *miR-182-5p*, *miR-96-5p* and *U6 snRNA* are mmu482690_mir, mmu481559_mir, mmu481282_mir and 001973 respectively) and M-MLV reverse transcriptase, and quantified using a TaqMan miRNA probe, *U6 snRNA* was used as the reference gene. For other miRNAs, cDNA was synthesized and quantified using Bulge-Loop miRNA qRT-PCR Starter Kit (1 RT primer and a pair of qPCR primers for each set specific for *miR-124*, *miR-9*, *miR-23a* were designed by RiboBio), *U6* was used as an internal control. For mRNAs and lncRNAs detection, cDNA was synthesized with M-MLV reverse transcriptase and random primers, and quantified using SYBR Green Master Mix (Roche). *Gapdh* was used as the reference gene. The relative expression of RNAs was calculated by using equation 2^−ΔΔCT^. The sequences of the sense and antisense primers are listed in Table.

**Table.** Primers Used in the qRT-PCR Experiments

	Forward	Reverse
*Ago2*	CGCGTCCGAAGGCTGCTCTA	TGGCTGTGCCTTGTAAAACGCT
*Tug1*	GAGACACGACTCACCAAGCA	AGGGTAGAGAGTATGAATAGC
*Rncr2*	AGAGACCTCTGCCACAGACTT	CGCGATCGCGGACCTGGCGCC
*Meg3*	GTGGTCTGGGGGCAGCCCCTT	CAGAGCCCACATCAGCCATCT
*Vax2os*	CCAGGAGCCGCCTAGCCACTC	TGGGAACATGGGTGCCTCGCC
*Six3os*	GCAGCAGGGACTTCAGGCGAC	CAGTGCGCTACCAGCACAGGG
*Gapdh*	AGGTCGGTGTGAACGGATTTG	TGTAGACCATGTAGTTGAGGTCA

### Western Blotting

Retinas were isolated, collected, and lysed in lysis buffer containing 1 × phenylmethanesulfonyl fluoride. Protein was extracted and quantified using a BCA protein assay kit (Invitrogen). Proteins were separated using SDS-PAGE and then analyzed by anti-Ago2 (1:1000, Abcam, ab186733) and anti-GAPDH (1:1000, KangChen Bio-tech, KG-5G4).

### Statistical Analysis

The values shown in the graphs represent averages of several independent experiments and the actual number of samples for each experiment stated in the figure legends. Data are presented as means ± SEM. The Mann–Whitney *U* test and 2-way ANOVA were adopted.

## Results

### Temporal Expression Pattern of *Ago2* During Retinal Development

To determine the Ago2 expression pattern during retinal development, we performed Western blots of E18.5, P1, P7, P14, P30, and P60 retinas. We found that the expression of Ago2 proteins were stable from E18.5 to P14, and decreased gradually from P14 to adulthood (Supplementary Fig. S1A, B).

### Deletion of Ago2 in Mouse Retina

**Figure 1.**
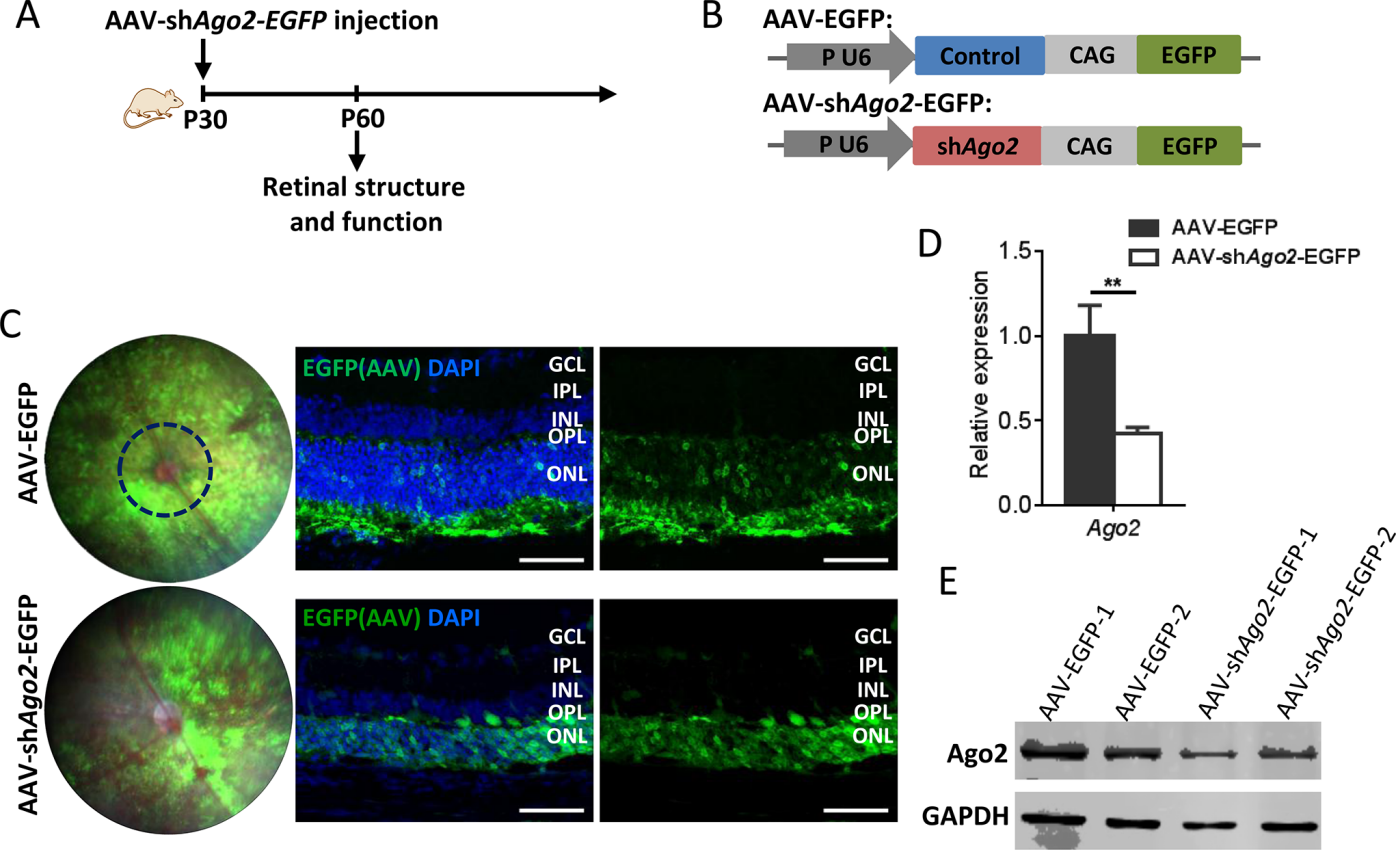
AAV-mediated silencing of Ago2 was introduced into the retina successfully. (**A**) Schematic representation of the experiments. AAV-sh*Ago2*-EGFP or AAV-EGFP vector was introduced into the subretinal space of mice at P30, retinal structure and function was analyzed at P60. (**B**) AAV-based *Ago2* shRNA and control AAV-EGFP driven by the pU6 promoter. (**C**) Fundus photography (*left*) and retinal cross-sections (*right*) of mice at P60 (*n* = 3). (**D**, **E**) qRT-PCR (**D**) and Western blot (**E**) of Ago2 expression in AAV-sh*Ago2*-EGFP-injected retinas compared with AAV-EGFP injected retinas at P60 (*n* = 3). The normalized values represent mean ± SEM. **P < 0.005; Mann–Whitney *U* test.

To assess the function of Ago2 in the retina, AAV-mediated silencing of Ago2 was introduced into the retina. We injected AAV-sh*Ago2*-EGFP and its control AAV-EGFP into the subretinal space of wild-type mice at P30 (Figs. 1A, B). Fundus photographs showed the absorption of AAVs at P60 (Fig. 1C, left) and cross-sections of the retina showed strong GFP signals in the ONL (Fig. 1C, right). Colocalization of GFP and Recoverin further verified that photoreceptor was infected successfully by AAVs (Supplementary Fig. S2). As expected, the qRT-PCR examination at P60 demonstrated a significant downregulation of *Ago2* expression (decreased to 42.70%) (Fig. 1D). Western blots showed a significantly decreased expression of Ago2 protein in mouse retina (Fig. 1E). These results suggested that Ago2 can be disrupted successfully in the photoreceptor cells.

### Deletion of Ago2 Impairs Retinal Structure

**Figure 2.**
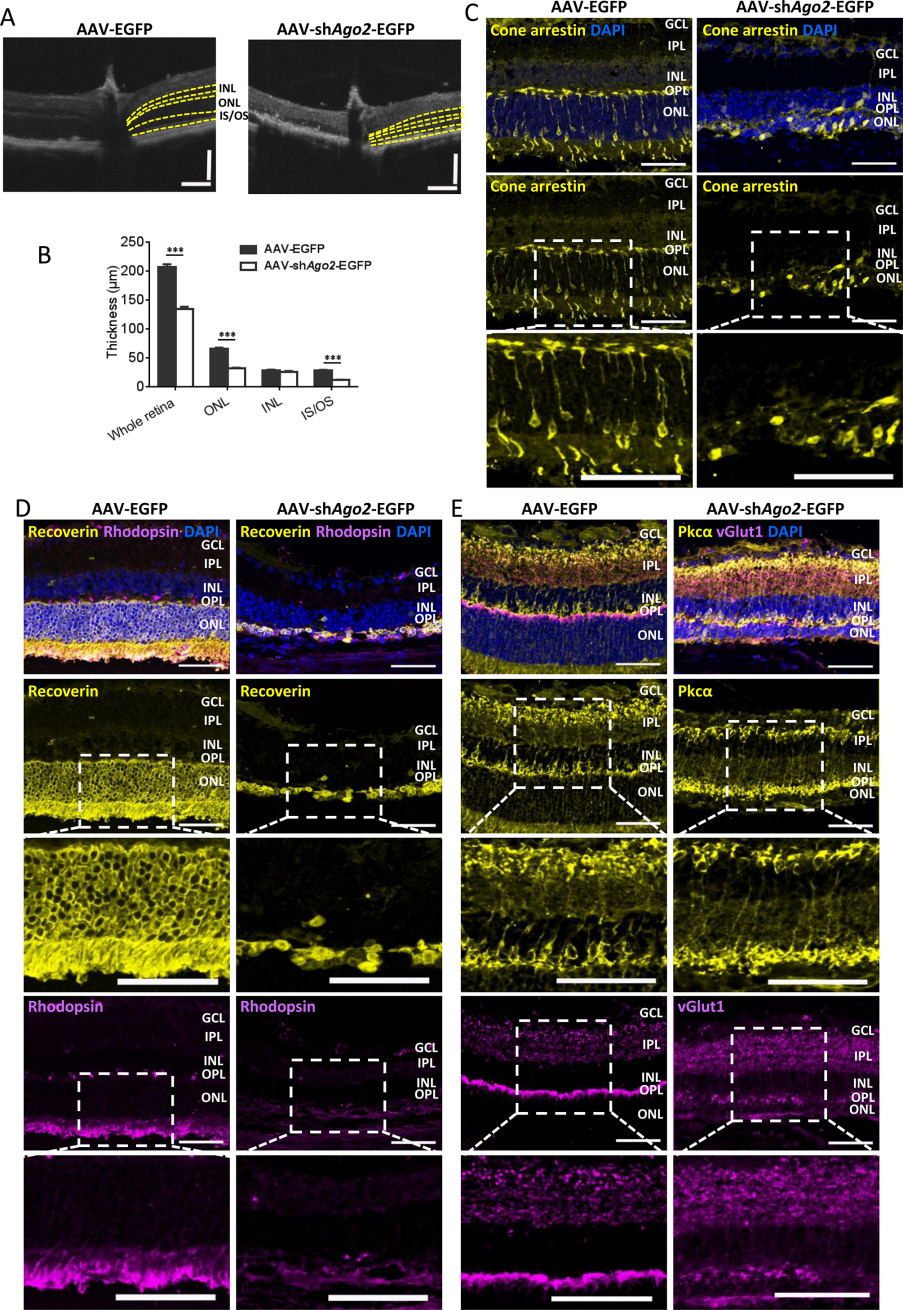
Silencing of Ago2 caused degenerative features in the photoreceptors. (**A**) SD-OCT of mice 30 days after AAV-sh*Ago2*-EGFP or AAV-EGFP subretinal injection (*n* = 10). (**B**) Quantification of the thicknesses of the whole retina, ONL, INL, and IS/OS at 400 µm location in reference to the optic nerve in A (*n* = 10). (**C**–**E**) Cone arrestin (**C**), Recoverin/Rhodopsin (**D**), and Pkcα/vGlut1 (**E**) immunostaining in retinas 30 days after AAV-sh*Ago2*-EGFP or AAV-EGFP subretinal injection (scale bar: 50 µm; *n* = 3). Separated channels and zoomed images of the immunostaining are shown. The normalized values represent mean ± SEM. ***P < 0.001; Mann–Whitney *U* test.

We first surveyed alterations in retinal morphology and structure after Ago2 deletion (Figs. 1, 2). Fundus photographs showed that no obvious abnormalities in the fundus were observed in AAV-sh*Ago2*-EGFP–treated retinas (Fig. 1C, left). Retinal thickness was also measured by SD-OCT. We found that the retina displayed a thinner IS/OS, ONL, and whole retina, whereas the inner nuclear layer (INL) seemed to be unaffected after Ago2 removing (Figs. 2A, B and Supplementary Fig. S3). Nuclei counting results showed that the ONL sharply decreased to 22.92% in AAV-sh*Ago2*-EGFP treated retina (Supplementary Fig. S4A). In addition, the TUNEL assay showed that apoptotic cells were clearly detected in the photoreceptor layer after Ago2 removing (Supplementary Fig. S4B).

To better understand these defects in retinal structure, we examined photoreceptors, bipolar neurons and synapses by immunostaining in P60 retinas (Figs. 2C–E). Immunostaining for Cone-arrestin, Rhodopsin, and Recoverin revealed that the photoreceptors were lost, with the ONL largely decreasing and the IS/OS almost disappearing (Figs. 2C, D), which is consistent with the results on OCT. Immunostaining for Pkcα showed that bipolar cells were relatively unaffected in Ago2-deficient retinas (Fig. 2E). Presynaptic termini, immunolabeled with vGlut1 (a protein located in synaptic vesicles in ribbon synapses), showed that synapses almost disappeared in the outer plexiform layer, but were unaltered in the inner plexiform layer (Fig. 2E). Immunolabeling with glial fibrillary acidic protein showed that the Müller glia (MG) responded to neuronal loss and became activated after Ago2 silencing (Supplementary Fig. S5A). Furthermore, immunofluorescence for cellular retinaldehyde binding protein and glutamine synthetase further showed that MG was not damaged obviously after Ago2 removing (Supplementary Fig. S5B, C). Taken together, these data suggest that the silencing of Ago2 led to retinal degeneration obviously and the damage mainly occurred in the outer retina layer.

### Depletion of Ago2 Results in Retinal Dysfunction

**Figure 3.**
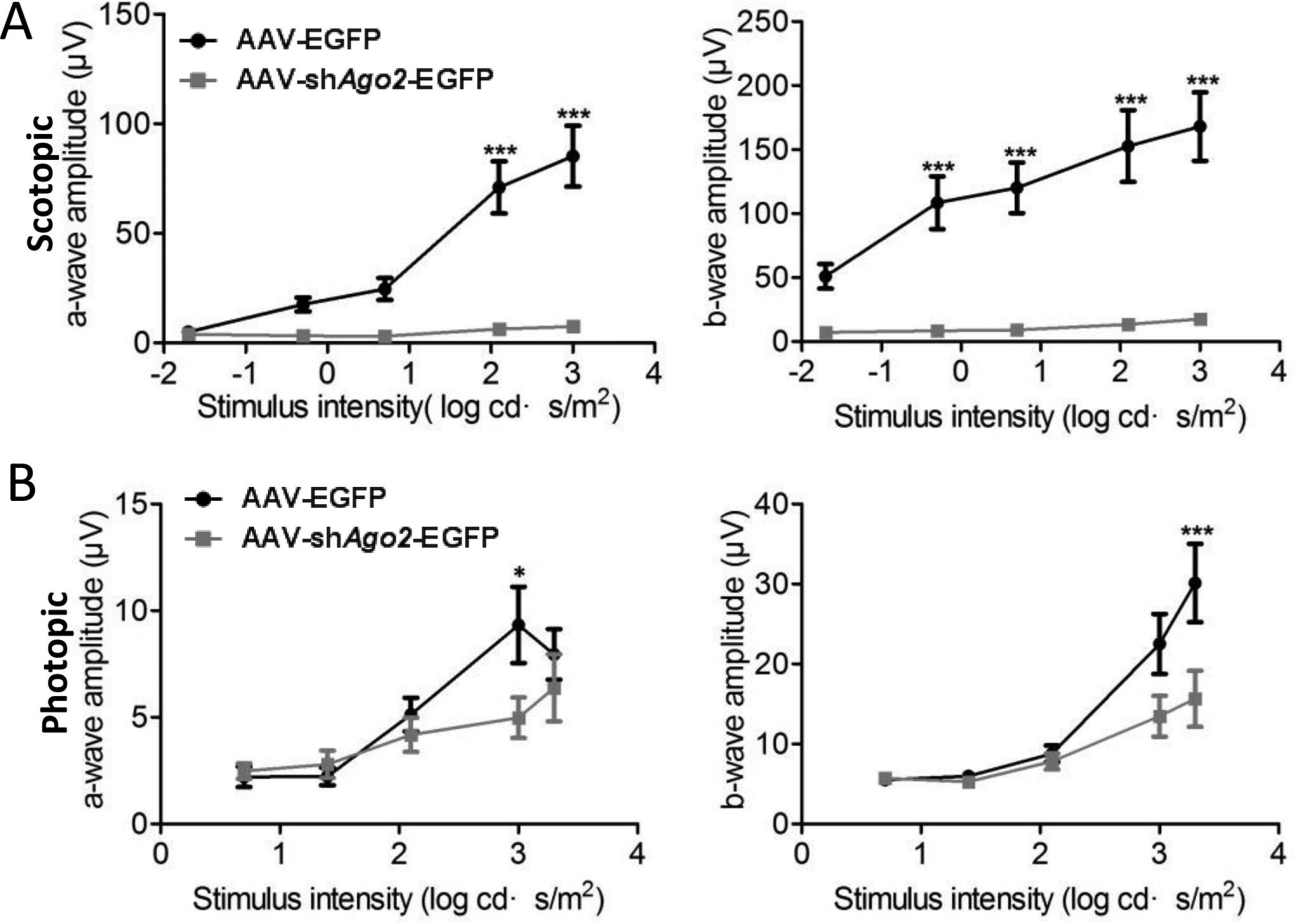
Silencing of Ago2 led to an abolished scotopic response and compromised photopic response. ERG recording to assess the rod (**A**) and cone (**B**) photoreceptors 30 days after AAV-sh*Ago2*-EGFP or AAV-EGFP subretinal injection (*n* = 10). The normalized values represent mean ± SEM. *P < 0.05; ***P < 0.001; Mann–Whitney *U* test.

Next, we evaluated retinal function in Ago2-deficient retinas at P60 by ERG. Of note, Ago2-deficient mice displayed an abolished scotopic response lacking a- and b-wave amplitudes at each stimulus intensity (Fig. 3A). This finding indicates that rod cells were severely damaged, which is consistent with the results of the OCT and immunostaining assays. We also assessed cone function in Ago2-deficient mice. The photopic response of mice seemed to show decreased a- and b-wave amplitudes progressively with increasing stimulus intensity (Fig. 3B). The relatively mild changes in photopic response suggested that the effect of Ago2 was mainly on rod cells. These findings confirmed that Ago2 is indispensable for retinal function.

### Deregulated Noncoding RNAs in the Retina After Ago2 Deletion

**Figure 4.**
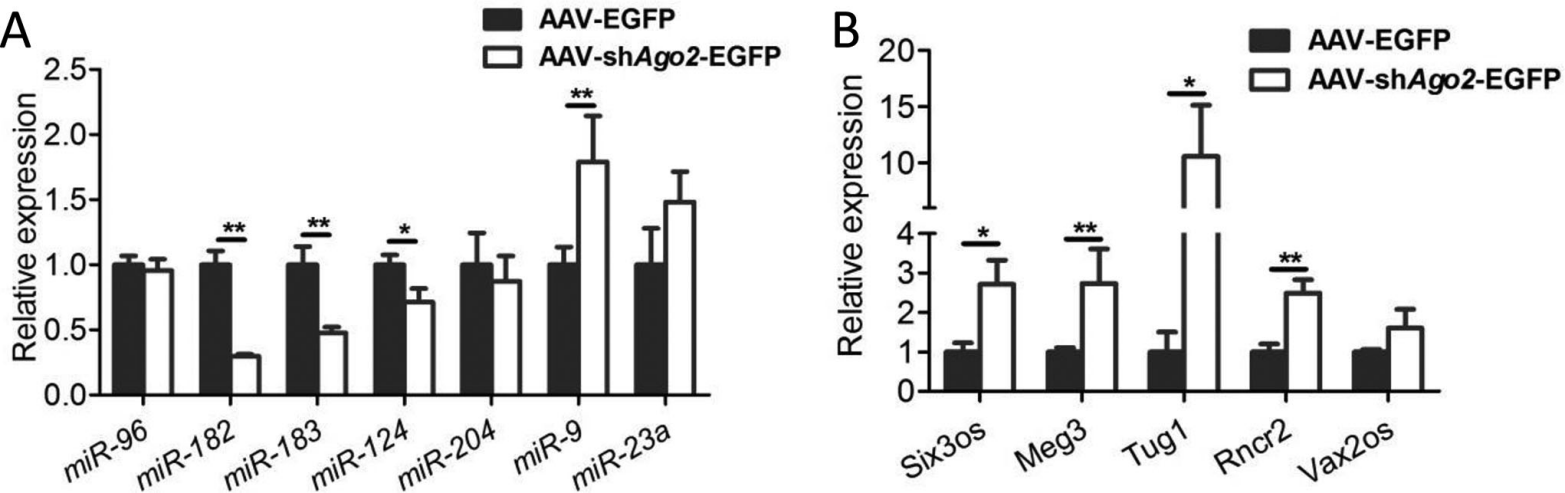
Silencing of Ago2 led to dysregulation of noncoding RNAs. (**A**, **B**) detection of the expression of miRNAs by qRT-PCR expressed in the retina (**A**) and lncRNA (**B**) in AAV-sh*Ago2*-EGFP and AAV-EGFP treated retinas at P60 (*n* = 4). The normalized values represent mean ± SEM. *P < 0.05; **P < 0.005; Mann–Whitney *U* test.

As the major player of RISC, the Ago2 protein directly binds to miRNA^39^^,^^40^ and then mediates miRNA function.^41^ Ago2 disruption may lead to dysregulation of genes in the retina, followed by severe retinal degeneration, especially in the photoreceptors. To explore the subsequent consequences of Ago2 deletion on a molecular level, we measured the retina-associated miRNAs of interest^23^^–^^31^^,^^42^^,^^43^ in Ago2-deficient retinas. Our results showed that some neuronal miRNAs, such as *miR-183C*,^23^^–^^26^ *miR-204*,^27^^,^^28^ and *miR-124*^29^ mainly expressed in ONL and/or INL, were decreased. However, the non-neuronal miRNAs, such as *miR-9* and *miR-23a*,^30^^,^^31^ which are highly expressed in MG, were increased (Fig. 4A). These results further verified that some changes happened in the MG, which is consistent with the results in immunostaining assay.

We then investigated the expression of retina-associated lncRNAs reported previously,^15^ among which, *Tug1*,^32^ *Rncr2*^33^^,^^34^ and *Six3os*^44^ are expressed predominantly in the INL and ganglion cell layer, and *Vax2os* expression is restricted to the ONL of the ventral retina.^45^ We found that most lncRNAs were found to be upregulated after Ago2 silencing (Fig. 4B), indicating that the changes at the molecular level had happened, even though the retinal structure in the inner retina was relatively intact. These results further indicated that Ago2 is essential for retinal maintenance.

### Overexpression of Ago2 Led to Retinal Degeneration

**Figure 5.**
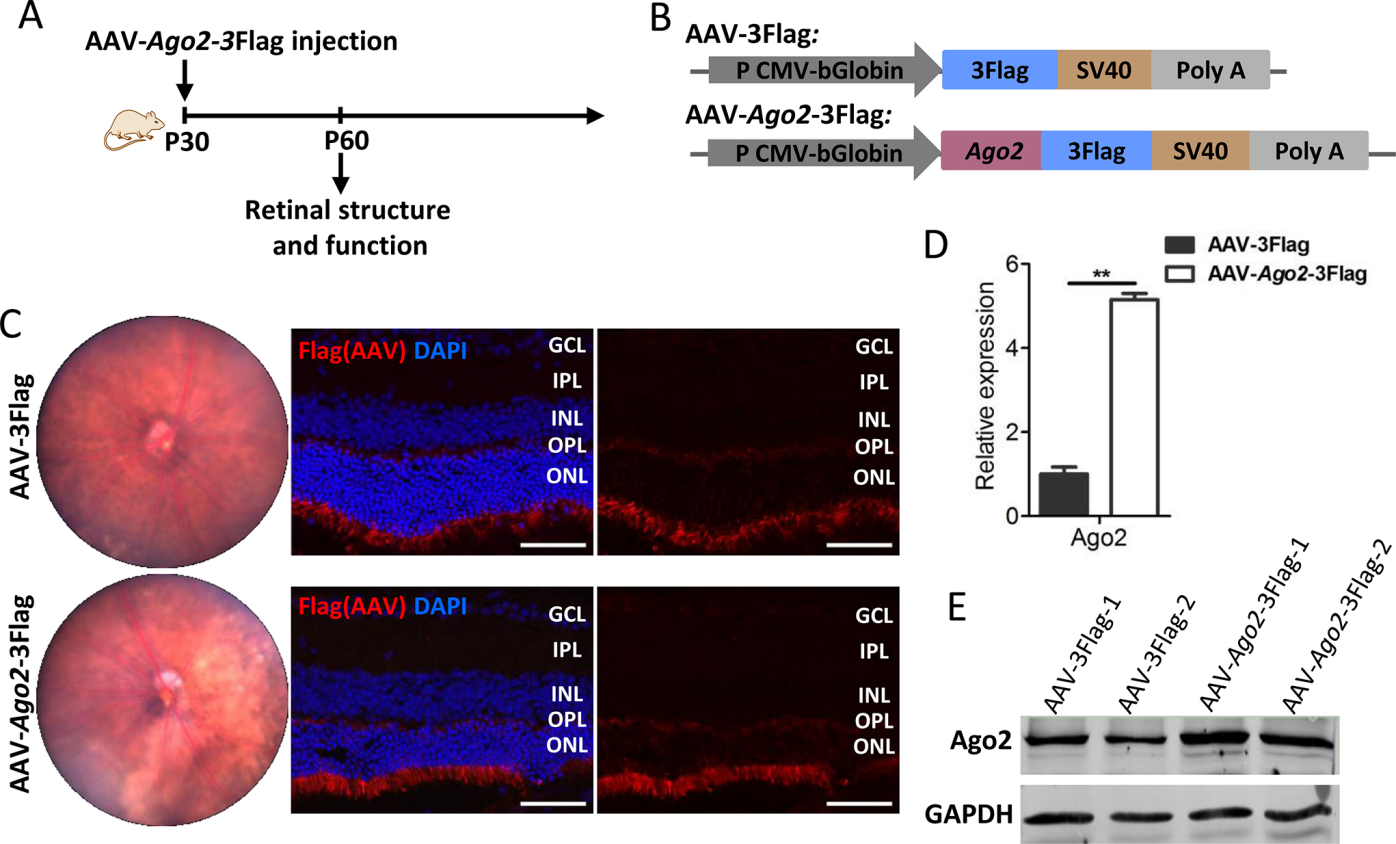
AAV-mediated overexpression of Ago2 was introduced into the retina successfully. (**A**) Schematic representation of the experiments. AAV-*Ago2*-3Flag or AAV-3Flag vectors were introduced into the subretinal space of mice at P30, retinal structure and function were analyzed 30 days after the AAVs injection. (**B**) AAV-based *Ago2* overexpression and control AAV-3Flag driven by the pCMV promoter. (**C**) Fundus photography (*left*) and retinal cross-sections (*right*) of mice 30 days after AAV-*Ago2*-3Flag or AAV-3Flag subretinal injection (*n* = 3) (**D**, **E**) qRT-PCR (**D**) and Western blot (**E**) of *Ago2* expression in retina 30 days after AAV- *Ago2*-3Flag or AAV-3Flag subretinal injection (*n* = 3). The normalized values represent mean ± SEM. **P < 0.005; Mann–Whitney *U* test.

It has been reported that Ago2 could bind and stabilize mature miRNAs, and thereby enhance miRNAs abundance and guide more mRNA silencing.^34^ Whether the overexpression of Ago2 in mouse retina leads to dysregulation of retinal miRNAs and retinal impairment remains unknown. Therefore, we injected AAV-*Ago2*-3Flag and its control AAV-3Flag into wild-type mice through subretinal injection at P30 (Figs. 5A, B). Retinal cross-sections displayed the successful expression of the Flag in the outer retina (Fig. 5C, right). Colocalization of Flag and Recoverin further verified that Ago2 is expressed in photoreceptor, mainly in IS/OS, after AAVs subretinal injection (Supplementary Fig. S6). Western blot and qRT-PCR results at P60 showed that Ago2 increased significantly in the AAV-*Ago2*-3Flag group (Fig. 5D, E). These results indicated that Ago2 can be successful overexpressed in the photoreceptor via AAV-mediated delivery.

**Figure 6.**
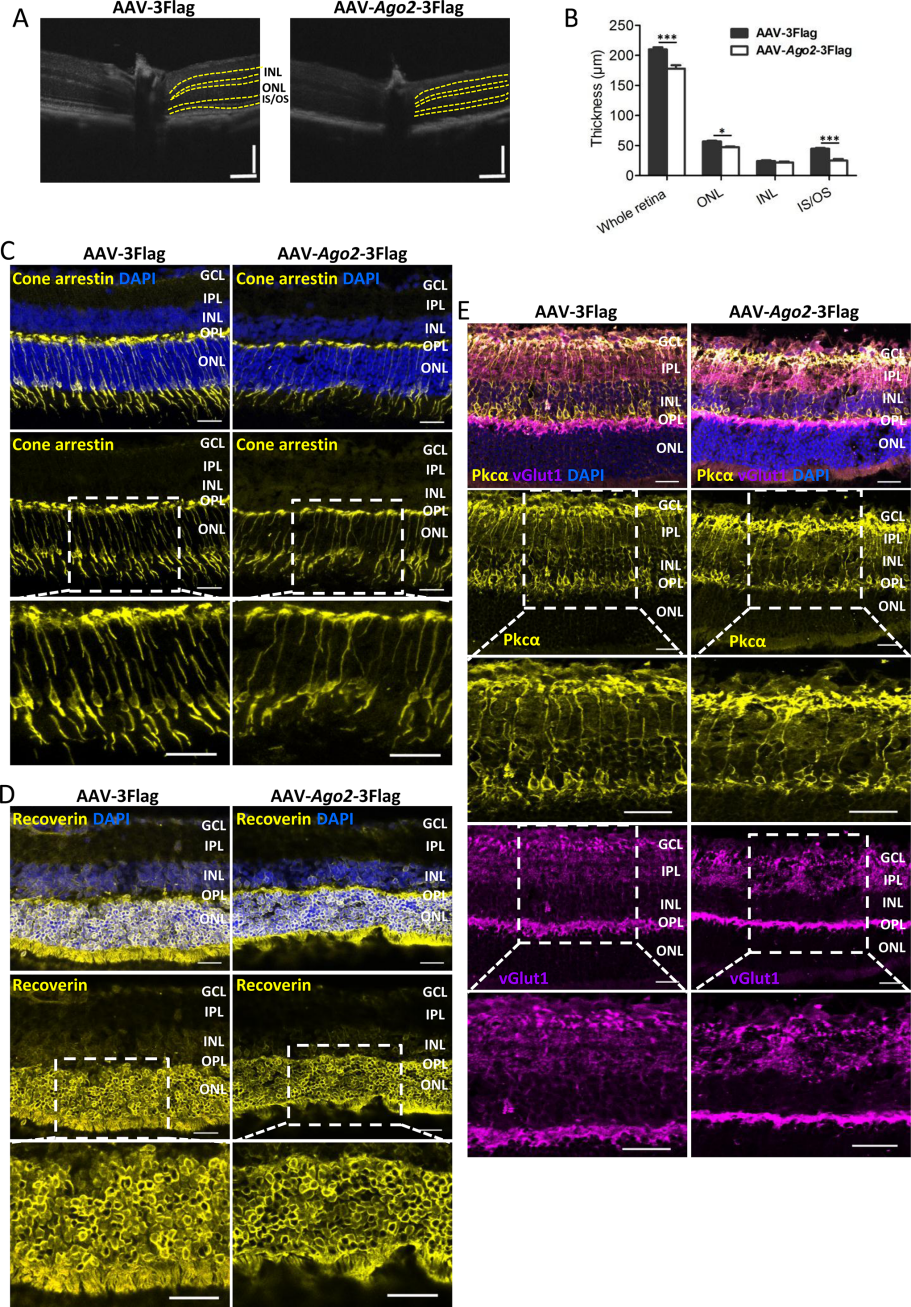
Overexpression of Ago2 led to a decreased retinal thickness and decreased photoreceptors. (**A**) SD-OCT of mice 30 days after AAV-3Flag or AAV-*Ago2*-3Flag subretinal injection (*n* = 10). (**B**) Quantification of the thicknesses of the whole retina, ONL, INL, and IS/OS at the 400 µm location in reference to the optic nerve in A (*n* = 10). (**C**–*E*) Cone arrestin (**C**), Recoverin (**D**), and PKCα/vGlut1 (**E**) immunostaining of retina 30 days after AAV-*Ago2*-3Flag or AAV-3Flag subretinal injection (scale bar: 25 µm, *n* = 3). Separated channels and zoomed images of the immunostaining are shown. The normalized values represent mean ± SEM. *P < 0.05; ***P < 0.001; Mann–Whitney *U* test.

However, no obvious abnormalities fundus was observed in AAV-*Ago2*-3Flag group (Fig. 5C, left). OCT results showed that the whole retina, ONL, and IS/OS were generally thinner in the AAV-*Ago2*-3Flag–treated retina. However, the INL seemed to be unaffected (Fig. 6A, B and Supplementary Fig. S7). This finding is consistent with the results in Ago2 deletion group. In addition, nuclei counting results showed that the ONL decreased to 53.46% mouse retina after Ago2 overexpression (Supplementary Fig. S4B).

Furthermore, immunostaining for Cone-arrestin, Recoverin, Pkcα, and vGlut1showed that architecture defects mainly happened in the outer retina and that the inner retina remains relatively intact after Ago2 overexpression (Figs. 6C–E). To investigate the MG in mouse retina after Ago2 overexpression, immunostaining for glial fibrillary acidic protein, cellular retinaldehyde binding protein, and glutamine synthetase were performed (Supplementary Fig. S8A–C). We found that MG was not damaged, but became activated in AAV-*Ago2*-3Flag treated retina. The overexpression of Ago2 led to retinal degeneration similar to that of the AAV-sh*Ago2*-EGFP–treated retina. However, the impairment after Ago2 overexpression was less severe.

**Figure 7.**
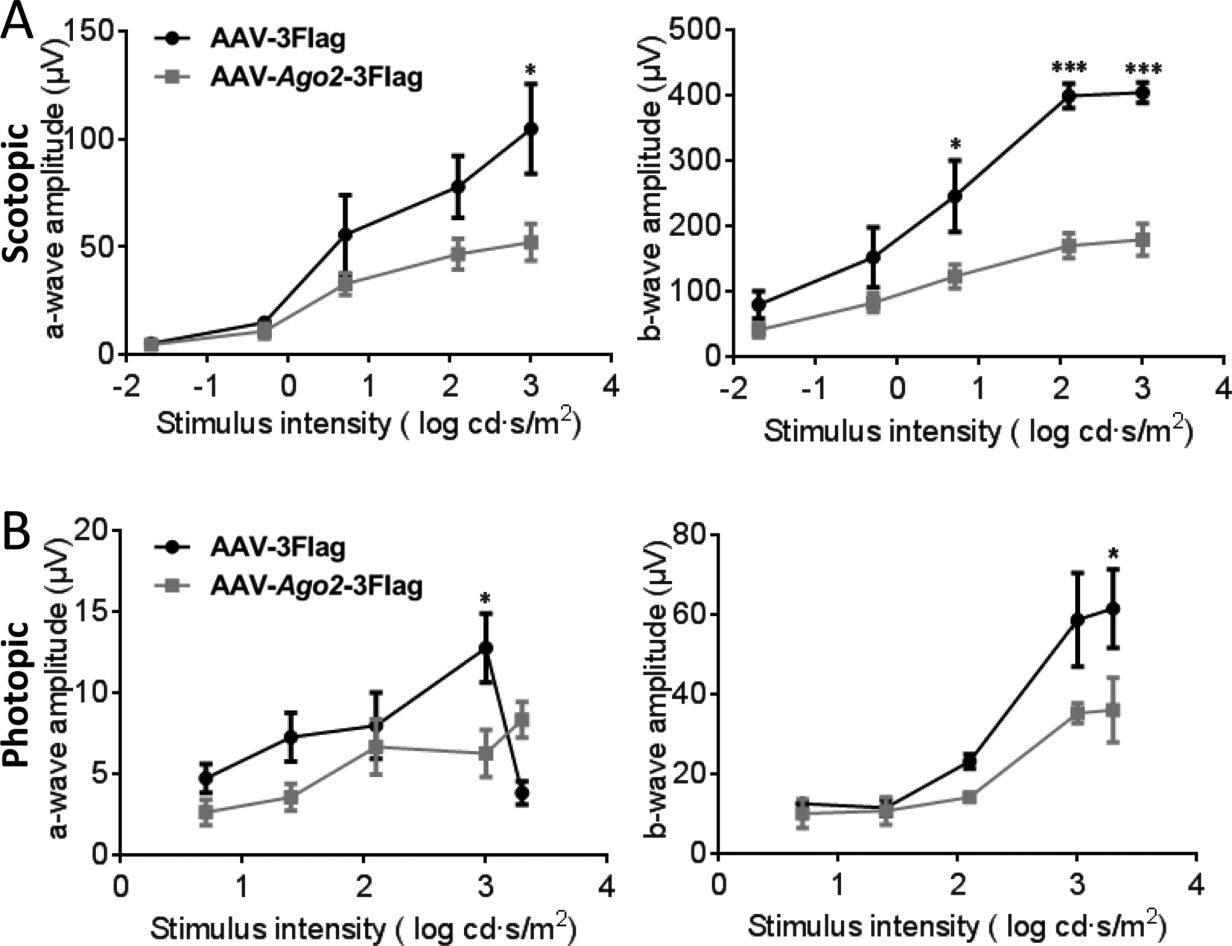
Overexpression of Ago2 led to compromised scotopic and photopic responses. ERG recording to assess the function of rod (**A**) and cone (**B**) photoreceptors 30 days after AAV-*Ago2-*3Flag or AAV-3Flag subretinal injection (*n* = 3). The normalized values represent mean ± SEM. *P < 0.05; ***P < 0.001; Mann–Whitney *U* test.

**Figure 8.**
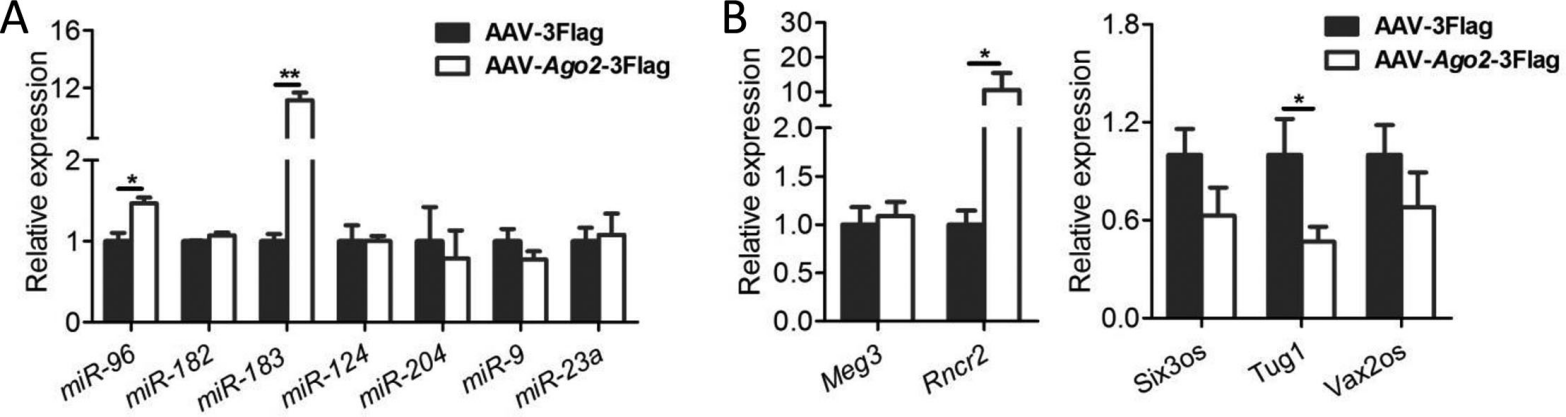
Overexpression of Ago2 led to dysregulation of noncoding RNAs. (**A**, **B**) RT-PCR detection of the expression of retinal-associated miRNA (**A**) and lncRNA (**B**) in retina after AAV-*Ago2-*3Flag or AAV-3Flag subretinal injection (*n* = 4). The normalized values represent mean ± SEM. *P < 0.05; **P < 0.005; Mann–Whitney *U* test.

Similar to the Ago2 silencing experiment, overexpression of Ago2 mainly affected the scotopic ERG response (Figs. 7A, B), indicating that the overexpression of Ago2 is detrimental to photoreceptors. In contrast, in the case of Ago2 overexpression, some retina-associated noncoding RNAs were either increased or decreased (Figs. 8A, B). These findings confirmed that steady-state Ago2 expression is indispensable for retinal function and maintenance.

## Discussion

We disrupted Ago2 expression in the retina by introducing AAV-sh*Ago2*-EGFP or AAV-*Ago2*-3Flag into the subretinal space of mice. As expected, silencing or overexpression of Ago2 led to a decreased retinal thickness and ERG response. And, the retinal degeneration caused by Ago2 silencing was more serious than Ago2 overexpression. This phenomenon is similar to a previous finding.^23^ Furthermore, retina-associated noncoding RNAs were abnormally expressed after Ago2 interruption. These results suggest that Ago2 homeostasis is essential for maintaining the normal morphology, structure, and function of mouse retina.

### The Effect of Ago2 Is Mainly on Photoreceptors

OCT and immunostaining assays showed that silencing of Ago2 resulted in severe defects in the outer retina, and the inner retina thickness remained relatively normal, indicating that retinal degeneration mainly occurred in the photoreceptors first and then extended to the inner retina.

ERG results showed that a-wave amplitudes were decreased in Ago2-deficient mice, especially in the scotopic response, and an immunostaining assay also showed decreased signals of Recoverin and absent signals of Rhodopsin. These results indicated that the effect of Ago2 was mainly on the photoreceptor layer. Compared with cones, rods were more susceptible to Ago2 deletion. This phenomenon may be due to the large portion of rods in the photoreceptors. In contrast, it has been reported that subretinally injected AAV vectors displayed a preference for rods as the photoreceptor matured. So rods were disrupted first and, then, loss of the outer segments of rod could make the AAV display a preference for cones.^46^ This finding also explained the decreased photopic response in our study. Considering the lack of changes in bipolar cells, we thought the decreased b-wave amplitude was mainly due to the decreased a-wave amplitude.

### Subretinal Injection Versus Intravitreal Injections

For the 2-way injections, subretinal injection usually has a direct effect on the resident cells and tissues in the subretinal space, such as photoreceptors.^47^ However, it could cause retinal detachment, so it required greater injection stability and proficiency. Intravitreal injection always targets the inner retina, such as the ganglion cells. Compared with subretinal injection, intravitreal injection is a simpler and less invasive technique. It could deliver a greater vector volume.^48^

The direct damage to photoreceptors in our results may be due to the subretinal injection method, which can directly affect photoreceptors. To investigate the effect of Ago2 on the inner retina, AAV-sh*Ago2*-EGFP and AAV-*Ago2*-3Flag were introduced into the retina via intravitreal injection at P30. Retinal cross-sections showed the successful absorption of AAVs by the inner retina at P60, especially the ganglion cell (Supplementary Figs. S9A, S10A). However, the intravitreal transduction efficiency is much lower than subretinal injection. Fundus and OCT results showed that silencing or overexpression of *Ago2* did not alter the fundus and retinal thickness obviously (Supplementary Fig. S9B, C and Supplementary Fig. S10B, C). Interestingly, the Pang group found that intravitreal injection of phenylalanine (Y-F) capsid mutant AAV8-mediated CNGA3 expression can restore cone function in CNGA3^−/−^/Nrl^−/−^. They also compared subretinal injection and intravitreal injection and found that both of these 2 methods could cause positive CNGA3 expression in cones.^49^ This outcome may be due to the different serotypes of AAV displaying different transduction profiles for retinal cells.^48^

### Retinal Degeneration Caused by Ago2 Disruption

As the key regulator in miRNA homeostasis, Ago2 binds to miRNAs directly and becomes a component of the ribonucleoprotein complex that regulates miRNA function.^50^^,^^51^ Ago2 interruption led to a decreased threshold for miRNA-mediated retina-associated gene silencing, which caused retinal degeneration and was followed by the abnormal expression of neuronal and non-neuronal miRNAs. In contrast, a specific role of Ago2 in miRNA maturation was identified.^37^ Ago2 cleaves the pre-miRNA to a novel intermediate (ac-pre-miRNA) in certain miRNA biogenesis. Ago2 deletion decreased the expression of some certain mature endogenous miRNAs.^12^^,^^37^ We thought that Ago2 interruption in our study decreased some miRNAs directly and that the dysregulation of miRNAs further led to the retinal degeneration.

In addition, the phenotypes caused by Ago2 disruption were similar to that reported in *miR-183/96* double KO model.^23^^,^^24^ However, the impact of Ago2 was mainly on rods, the impact of *miR-183/96* was mainly on cones.^23^ The defects in the *miR-182* KO^25^ or *miR-183* KO^26^ mouse model are slighter than in the Ago2 knockdown mouse model; *miR-182* or *miR-183* deletion led to a decreased ERG response but did not affect the retinal morphology. Target disruption of *Rncr3*, the dominant source of *miR-124a*, caused reduced cones.^29^ The deletion of *miR-211* results in progressive cone dystrophy, but does not affect the function of rods.^52^ It has been reported that Dicer deletion from the MG resulted in decreased miRNAs and damaged retinal architecture and function.^30^ Decreased *miR-9* and upregulated target Bcan is responsible for disorganized MG phenotype. The increased *miR-9* after Ago2 silencing also led to MG activation in our study, implying that miRNAs are essential for glia homeostasis and retinal architecture.

### Studies About Factors Involved in miRNA Biogenesis

Among these factors involved in miRNA biogenesis and function, Ago2, Dicer, and Dgcr8 have been reported to be absolute requirement for mouse development, and disruption of Ago2, Dicer, or Dgcr8 resulted in early embryonic death.^10^^,^^53^^,^^54^

Dicer-null mice have been reported to die at E7.5.^53^ To decipher the function of Dicer in the retina, different conditional Dicer-null mice models have been generated using Chx10-Cre,^16^ αPax6-Cre,^17^^,^^18^ Dkk3-Cre,^19^ Rx-Cre,^20^ D4-Cre,^21^ and Dct-Cre ^22^ transgenic mice. These mouse models showed varying degrees of miRNAs reduction and retinal malformation and degeneration. Dicer has also been implicated in producing other small RNAs.^55^ However, Drosha has been reported to be involved in preribosomal RNAs processing, possibly in a distinct protein complex.^56^ By contrast, Dgcr8 seems to be specific to miRNAs owing to its acts in recognizing the pre-miRNA.^57^ *Dgcr8*-null ES cells showed that Dgcr8 is essential for miRNA biogenesis and silencing of ES cell self-renewal.^54^

The embryonic–lethal phenotype caused by Ago2 may be because, compared with the other RISC components PACT and TRBP,^58^^,^^59^ Ago2 has an earlier function in RISC to facilitate miRNA biogenesis.^13^ Another study pointed out that the ability of Ago2 to assemble into catalytically active complexes might be critical for mouse development.^10^ Previous studies just focused on deletion of Ago2 in ES cells^10^^,^^11^ and conditional deletion of Ago2 in oocytes.^12^ Studies of Ago2 in retina were only about exploring the Ago2 funciton during bone marrow-derived stem cell exosomes for treating ocular disease.^60^^,^^61^ However, the real role of the Ago2 in retina remains unclear. In addition, vectors based on AAV are currently preferred tools for gene studies in the retina.^21^^,^^23^ It is more convenient, effective, and faster than gene KO animal models. Our study provided significant instruction for further deciphering the role of Ago2 by KO mouse model.

Taken together, our findings demonstrated that the silencing or overexpression of Ago2 in mice led to a severe retinal degeneration phenotype. And, for the first time, suggested that Ago2 homeostasis is essential for retinal maintenance and function.

## Supplementary Material

Supplement 1
