# Supplementary material for: Retinal Degeneration Caused by Ago2 Disruption
Source: Invest Ophthalmol Vis Sci. 2021 Sep 16;62(12):14. doi: 10.1167/iovs.62.12.14 (PMC8447045; doi:10.1167/iovs.62.12.14)
Supplement: Supplement 1 [file iovs-62-12-14_s001.pdf]

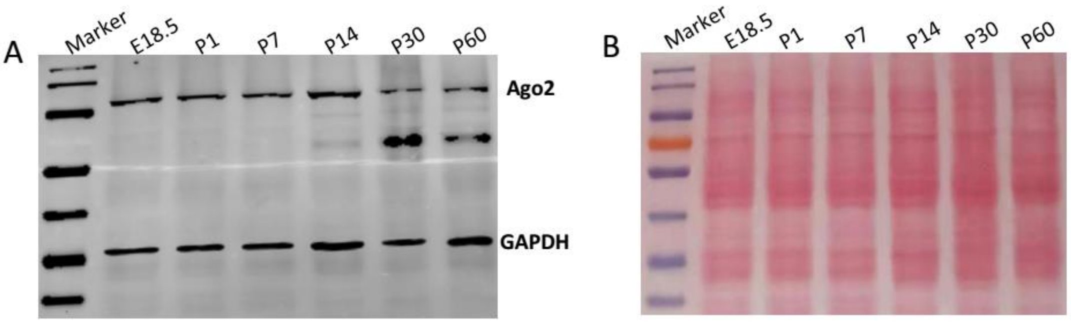

Figure S1. The expression pattern of Ago2 protein during retinal development. (A) Ago2 protein levels during retinal development assessed by western blot analysis. (n=3). (B) Ponceau S staining was used to quantify the total protein in western blot due to the different expression of reference gene GAPDH during retinal development (n=3).

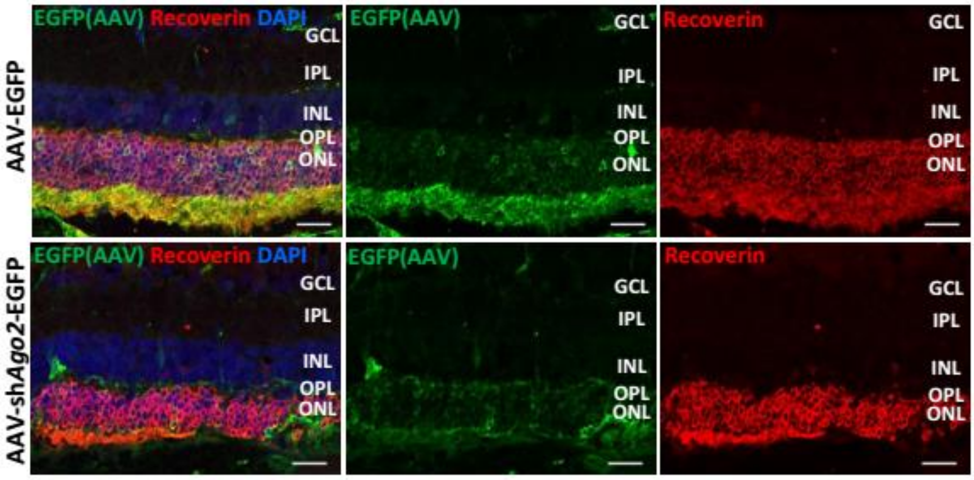

Figure S2. EGFP and Recoverin immunostaining of retina 30 days after AAV-sh*Ago2*-EGFP treatment (scale bar: 25 μm, n = 3). Separated channels are shown on the right.

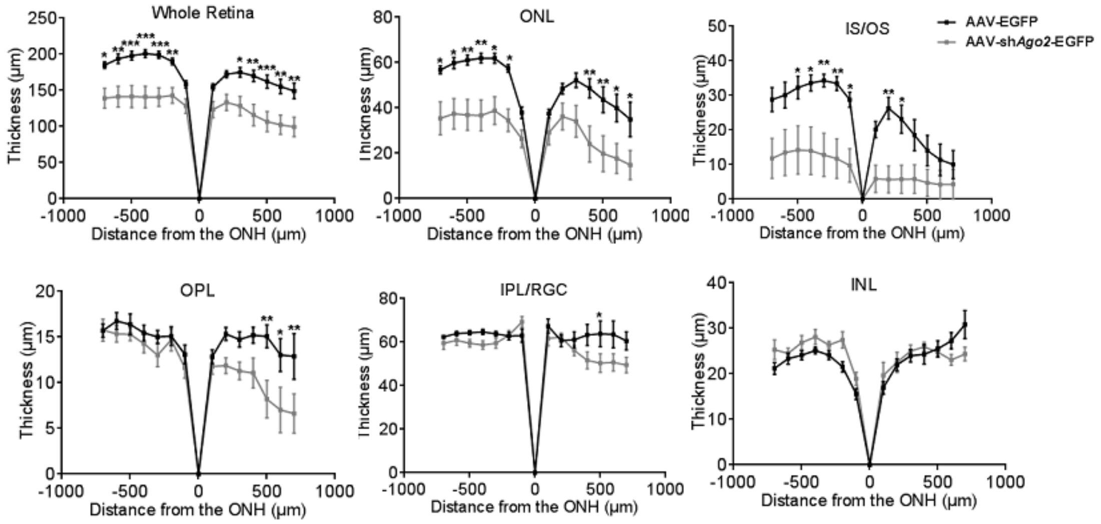

Figure S3. Quantification of the thickness of the whole retina, ONL, OS/IS, OPL, IPL/RGC and INL from SD-OCT images of mice 30 days after AAV-sh*Ago2*-EGFP or AAV-EGFP subretinal injection (n = 10). The normalized values represent mean ± SEM. *P < 0.05; **P < 0.005; ***P < 0.001; Mann-whitney test.

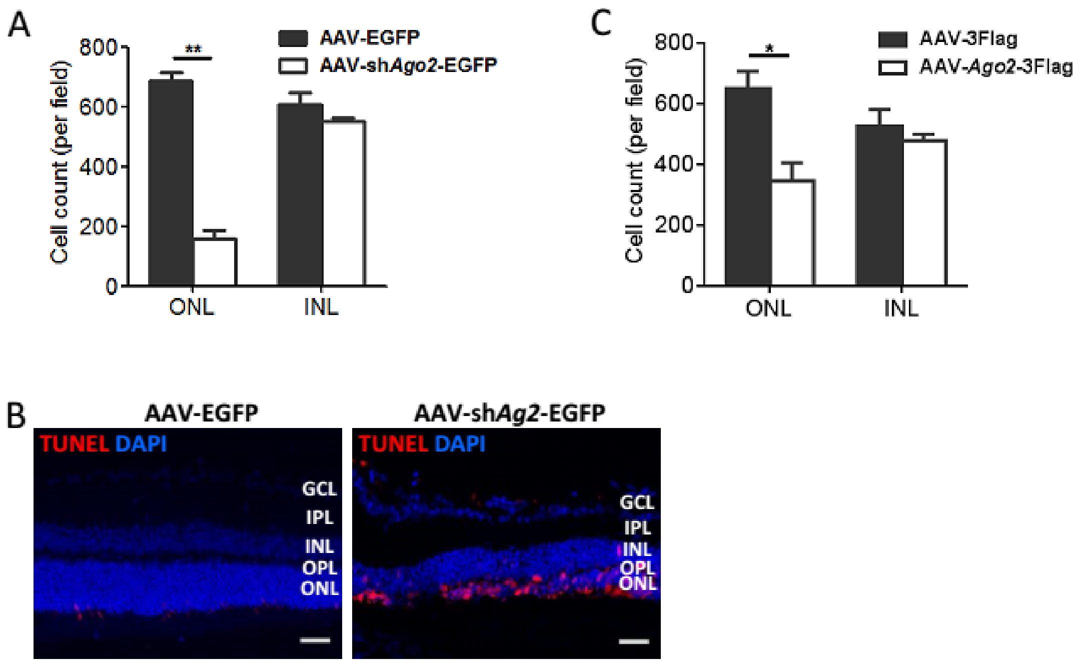

Figure S4. (A, B) Comparison of cell counts in ONL and INL 30 days after AAV-sh*Ago2*-EGFP or AAV-*Ago2*-3Flag subretinal injection (n=3). (C) Apoptosis of retinas was assessed in retinal sections using a TUNEL assay 30 days after AAV-sh*Ago2*-EGFP treatment (scale bar: 100). The red signals represented the

apoptotic cells. The normalized values represent mean ± SEM. *P < 0.05; **P < 0.005; Mann-whitney test.

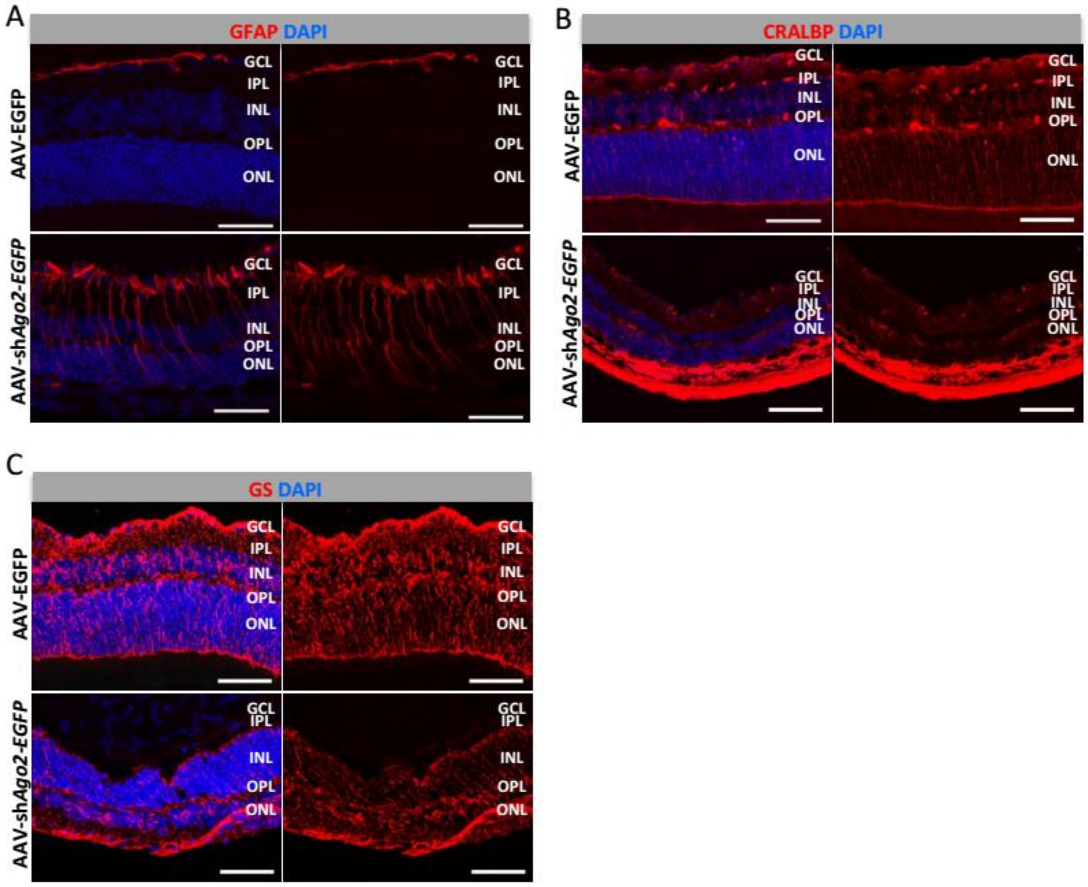

Figure S5. Immunostaining of MG in retinas 30 days after AAV-sh*Ago2*-EGFP treatment using the GFAP (A), CRALBP (B) and GS (C) antibodies (scale bar: 50 μm, n = 3). Separated channels are shown on the right.

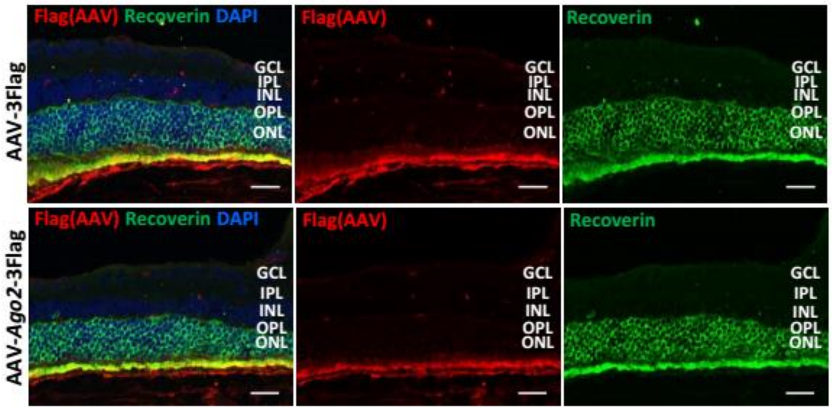

Figure S6. Flag and Recoverin immunostaining of retina 30 days after AAV-*Ago2*-3Flag or AAV-3Flag subretinal injection (scale bar: 25 μm, n = 3). Separated channels are shown on the right.

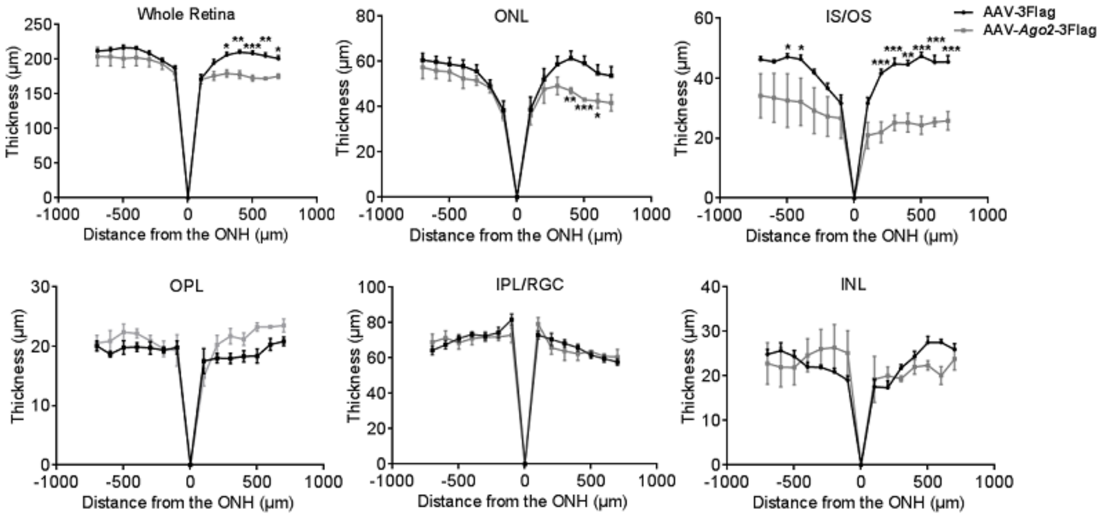

Figure S7. Quantification of the thickness of the whole retina, ONL, OS/IS, OPL, IPL/RGC and INL from SD-OCT images of mice 30 days after AAV- *Ago2*-3Flag or AAV-3Flag subretinal injection (n = 10). The normalized values represent mean ± SEM. *P < 0.05; **P < 0.005; ***P < 0.001; Mann-whitney test.

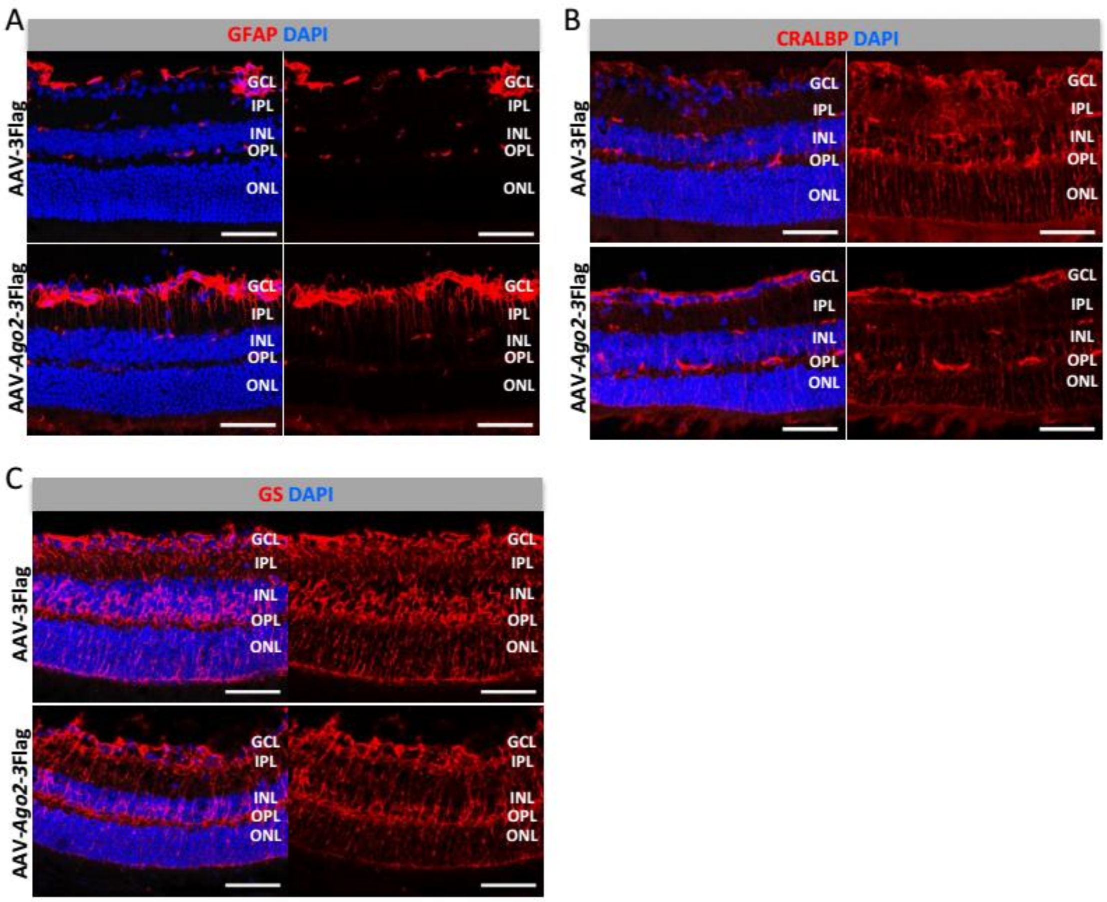

Figure S8. Immunostaining of MG in retina 30 days after AAV-*Ago2*-3Flag treatment using the GFAP (A), CRALBP (B) and GS (C) antibodies (scale bar: 50 µm, n = 3). Separated channels are shown on the right.

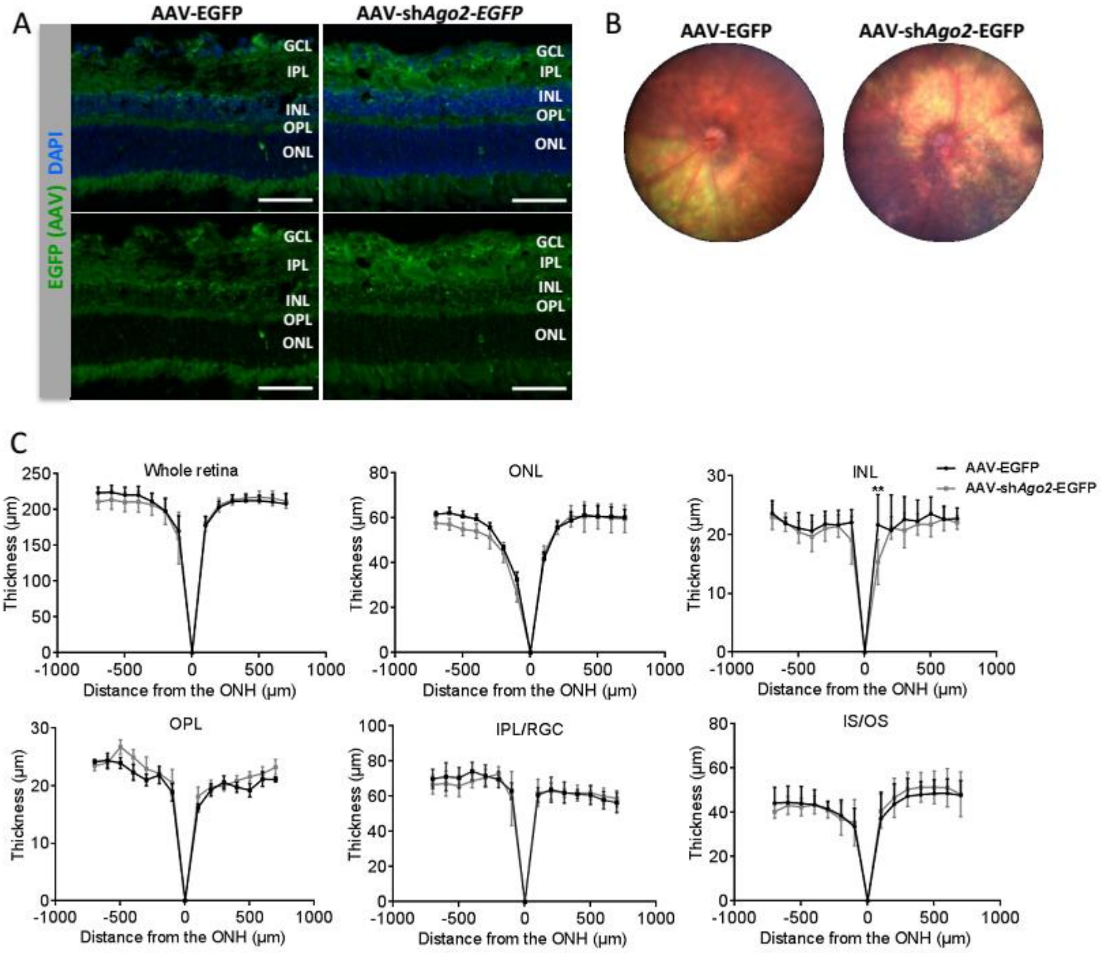

Figure S9. (A) Retinal cross section of mice 30 days after AAV-sh*Ago2*-EGFP intravitreal injection (n = 3). (B) Fundus photography of mice 30 days after AAV-sh*Ago2*-EGFP intravitreal injection (n = 3). (C) Quantification of the thickness of the whole retina, ONL, OS/IS, OPL, IPL/RGC and INL from SD-OCT images of mice after intravitreal injection of AAV-sh*Ago2*-EGFP (n=5). The normalized values represent mean ± SEM. **P < 0.005; Mann-whitney test.

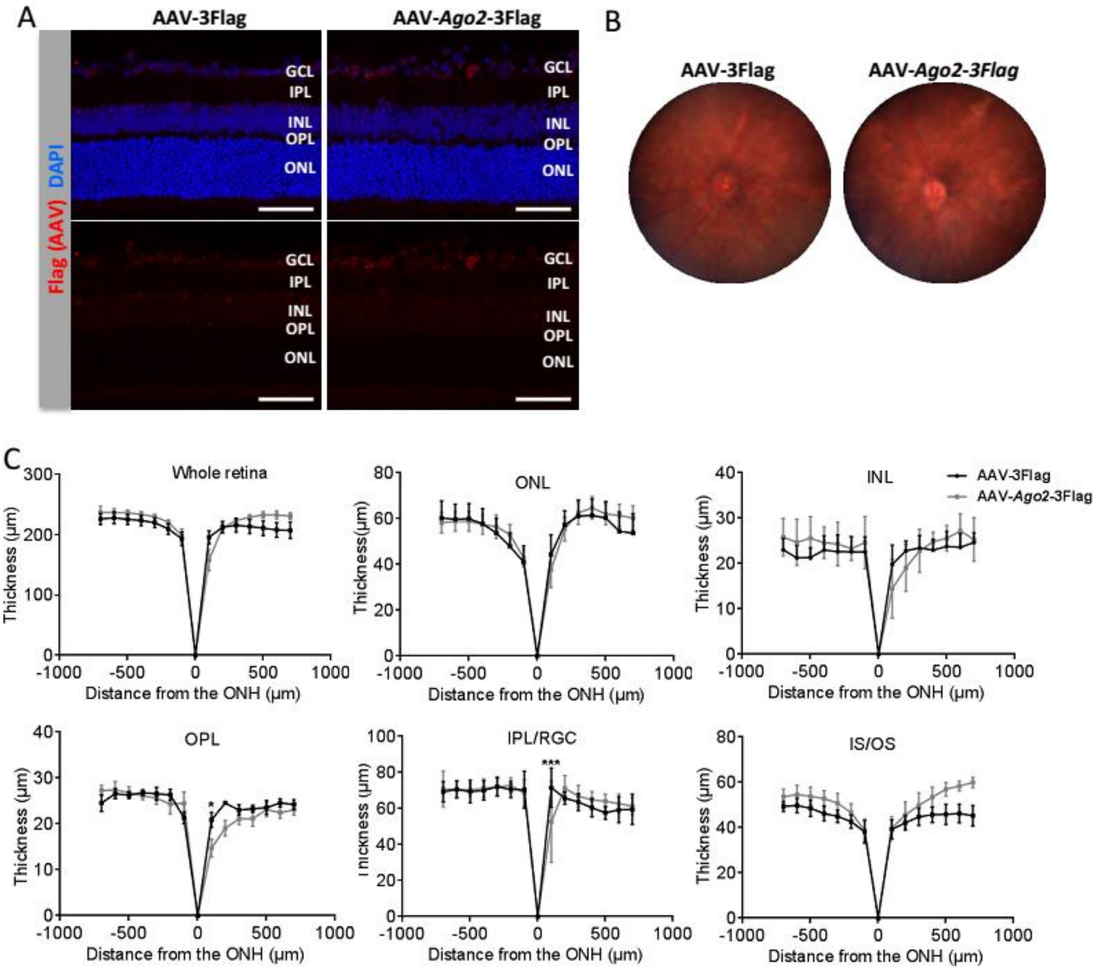

Figure S10. (A) Retinal cross section of mice 30 days after AAV-*Ago2*-3Flag intravitreal injection (n = 3). (B) Fundus photography of mice 30 days after AAV -*Ago2*-3Flag intravitreal injection (n = 3). (C) Quantification of the thickness of the whole retina, ONL, OS/IS, OPL, IPL/RGC and INL from SD-OCT images of mice after intravitreal injection of AAV-*Ago2*-3Flag (n=6). The normalized values represent mean ± SEM. *P < 0.05; ***P < 0.001; Mann-whitney test.
